# Effect of COVID-19 on gastrointestinal endoscopy practice: a systematic review

**DOI:** 10.1080/07853890.2022.2133163

**Published:** 2022-11-11

**Authors:** Mohamed H. Emara, Mariam Zaghloul, Muhammad Abdel-Gawad, Nahed A. Makhlouf, Mohamed Abdelghani, Doaa Abdeltawab, Aya M. Mahros, Ahmed Bekhit, Nitin S. Behl, Sadek Mostafa, Alejandro Piscoya, Sherief Abd-Elsalam, Mohamed Alboraie

**Affiliations:** a Hepatology, Gastroenterology and Infectious Diseases Department, Kafrelsheikh University, Kafr El-Sheikh, Egypt; b Hepatology, Gastroenterology, and Infectious Diseases Department, Al-Azhar University, Assiut, Egypt; c Tropical Medicine and Gastroenterology Department, Faculty of Medicine, Assiut University, Assiut, Egypt; d Department of Gastroenterology, Sharqia Health Directorate, Sharqia, Egypt; e Institute of Gastro and liver Diseases, Fortis Hospitals, Ludhiana, India; f Department of Internal Medicine, Al-Azhar University, Cairo, Egypt; g Department of Gastroenterology, Hospital Guillermo Kaelin De la Fuente – EsSalud, Lima, Peru; h Systematic Reviews and Meta-analysis, Clinical Practice Guidelines and Health Technology Assessments Unit, Universidad San Ignacio Loyola, Lima, Peru; i Tropical Medicine and Infectious Diseases Department, Tanta University, Tanta, Egypt

**Keywords:** Gastrointestinal endoscopy, COVID-19, pandemic, practice, SARS-Cov-2

## Abstract

**Background:**

Since the emergence of the novel corona virus (SARS-Cov-2) in the late 2019 and not only the endoscopy practice and training but also the health care systems around the globe suffers. This systematic review focused the impact of Corona Virus Disease (COVID-19) on the endoscopy practice.

**Methods:**

A web search of different databases combining different search terms describing the endoscopy practice and the COVID-19 pandemic was done. Articles were screened for selection of relevant articles in two steps: title and abstract step and full-text screening step, by two independent reviewers and any debate was solved by a third reviewer.

**Results:**

Final studies included in qualitative synthesis were 47. The data shown in the relevant articles were evident for marked reduction in the volume of endoscopy, marked affection of colorectal cancer screening, impairments in the workflow, deficiency in personal protective equipment (PPE) and increased likelihood of catching the infection among both the staff and the patients.

**Conclusion:**

The main outcomes from this review are rescheduling of endoscopy procedures to be suitable with the situation of COVID-19 pandemic in each Country. Also, the endorsement of the importance of PPE use for health care workers and screening of COVID-19 infection pre-procedure.

**Key messages**
- The data focussing Gastrointestinal Endoscopy and COVID-19 emerged from different areas around the globe. The data presented on the published studies were heterogeneous. However, there were remarkable reductions in the volume of GI endoscopy worldwide
- Staff reallocation added a burden to endoscopy practice
- There was a real risk for COVID-19 spread among both the staff and the patients

## Introduction

Since the emergence of the novel corona virus (SARS-Cov-2) out of Wuhan China in the late 2019 and its exponential spread around the globe and the health care systems suffers. This pandemic was named after the WHO as COVD-19, and it is obvious that no part of the globe was immune against the spread of this viral infection. During the pandemic, the health care systems including the endoscopy services in all countries were impaired through multiple mechanisms [1].

Gastrointestinal (GI) endoscopy is no more a complementary investigation in the management of GI disorders, it is rather an integral part of gut care and that is why endoscopy units are widely available among the health care facilities because the complete gut care without endoscopy is in vain. The services offered by GI endoscopy range from simple diagnostic, screening indications to therapeutic and sometimes lifesaving interventions. However, it was noticeable that the endoscopy practice as well as the scope of service was impaired by the Corona Virus Disease (COVID-19) pandemic. Furthermore, endoscopic procedures are aerosol generating; necessitate direct contact with the patients for a reasonable period of time, in addition to shedding the virus in the stool which ultimately increase the risk of infection to the endoscopy staff. Because of the deficiency in health care workers, reallocation of the staff added another burden to the endoscopy practice besides the deficiency in the protective equipments necessary to comply with infection control policies [1].

Consequently, different GI endoscopy societies formulated practice advices and recommendations concerning the endoscopy practice during the pandemic (Table 1) and some proposed strategies for the gradual resume of the full endoscopy activities after the pandemic is over [2–4]. However, it is obvious that the pandemic will remain for a while particularly with the difficulties facing the developed vaccines and the emergence of the mutant strains, which means that endoscopy practice, will not recover completely in the near future [5].

**Table 1.** Statements from different endoscopy societies during COVID-19 pandemic as for March 2020.

Asian Pacific Society for Digestive Endoscopy
Defer elective endoscopic procedures, while urgent endoscopies should be performed by strategically assigned staff to minimize concomitant exposure.
European Society of Gastrointestinal Endoscopy (ESGE)
Defer elective endoscopic procedures. The patient should be assessed for high versus low risk of GIT, while urgent endoscopies should be performed by strategically assigned staff to minimize concomitant exposure.
World Endoscopy Organization (WEO)
Postpone non-urgent examinations. Upper GI bleeding, foreign body/obstruction; acute cholangitis are not deferred.
American Society for Gastrointestinal Endoscopy (ASGE)
Postpone non-urgent examinations. Upper GI bleeding, foreign body/obstruction; acute cholangitis, care of cancer not postponed.
American College of Gastroenterology (ACG);
Postpone non-urgent examinations. Upper GI bleeding, foreign body/obstruction; acute cholangitis, care of cancer, prosthetic removals are not postponed.
Sociedad Interamericana de Endoscopia Digestiva (SIED); Canada/Central and Latin America
Postpone non-urgent examinations. Urgent procedures were not described.

**Table 2.** Different methods of triaging patients before endoscopy.

Study	History	CBC	CXR	CT	PCR
Al Mahtab et al. [12]	Yes	Yes	Yes		
Alboraie et al. [1]	Yes	Yes			Yes
An et al. [14]	Yes	Yes	Yes	Yes	Yes
Mauro et al. [15]				Yes	Yes
Becq et al. [9]					Yes
Chen et al. [16]					Yes
Dioscoridi and Carrisi [17]	Yes				
Ebigbo et al. [7]	Yes				15%
Forbes et al. [19]	Yes				
Garbe et al. [20]	Yes				Yes
Manes et al. [34]					Yes
Huang et al. [23]					Yes
Ikehara et al. [24]				Yes	Yes
Kim et al. [26]	Yes	Yes		Yes	Yes
Kushnir et al. [27]	Yes				Yes
Liu et al. [29]	Yes			Yes	Yes
Lui et al. [11]	Yes				
Maida et al. [32]	Yes				
Moreels [38]					Yes
Navaneethan et al. [39]	Temperature and COVID-19 symptoms				
O’Grady et al. [40]		Yes			Yes
Medas et al. [42]	Yes, by questionnaire				
Moraveji et al. [44]					Yes
Sobani et al. [45]	Yes	Yes	Yes	Yes	Yes
D'Ovidio et al. [08]	YES				
Belle et al. [47]					Yes
Zorniak et al. [49]	Yes				Yes

CBC: complete blood count; CXR: chest X ray; CT: computed tomography; PCR: polymerase chain reaction.

The data focussing the impact of COVID-19 on GI endoscopy were retrieved from different geographic locations and the data were heterogeneous regarding the type of studies, aims of the studies, quality of the studies; however, all were consistent in their conclusions confirming affection of the endoscopy practice during the pandemic. This systematic review focussed the impact of COVID-19 on the endoscopy practice analysing the available consistent evidence.

## Methods

### Search strategy and eligibility criteria

A systematic review about impact of COVID-19 on gastrointestinal endoscopy practice was conducted. We searched PubMed, Web of Science (WOS), Scopus, EBSCO, Wiley and WHO databases for relevant articles, using the following search terms ‘Coronavirus’[Mesh]) OR ‘SARS Virus’[Mesh] OR ‘COVID-19’ [Supplementary Concept]) OR ‘Pandemics’[Mesh]) AND ‘Endoscopy’ [Mesh]. We included any article contains original information regarding endoscopy status during the COVID-19 pandemic irrespective of the study type, population, or language.

### Screening, selection and data extraction

We excluded reviews, abstracts, duplicates, corresponding, commentaries, case reports and all non-original study types. All remaining articles retrieved from searching targeted databases and all manually added articles were screened for selection of relevant articles in two steps of screening: title and abstract step and full-text screening step, by two independent reviewers and any debate was solved by a third reviewer.

### Data synthesis and analysis

SPSS version 26 (IBM SPSS Inc., Chicago, USA) was used for data and subgroup analysis. The review was registered in the PROSPERO registry for systematic reviews (CRD42021229645).

### Ethics and informed consent of patients

Ethics committee approval and informed consent of patients were not required, as this study did not involve confidential patient information.

## Results

### Retrieved studies

**Figure 1.**
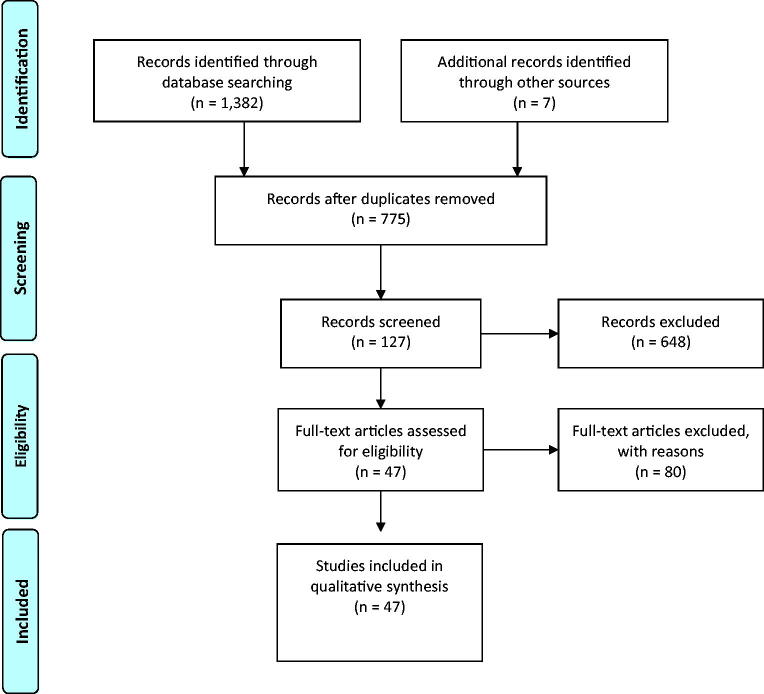
The PRISMA flow diagram for the retrieved studies.

Our electronic search retrieved 1382 eligible articles in addition to 7 articles added manually. After removal of duplicates, 775 articles submitted for screening. Out of them, 648 were excluded through title and abstract screening and out of the remaining 127 articles, 80 were also excluded after full-text screening. Final studies included in qualitative synthesis were 47 [1,2,6–50] as shown in the PRISMA flow diagram (Figure 1).

**Figure 2.**
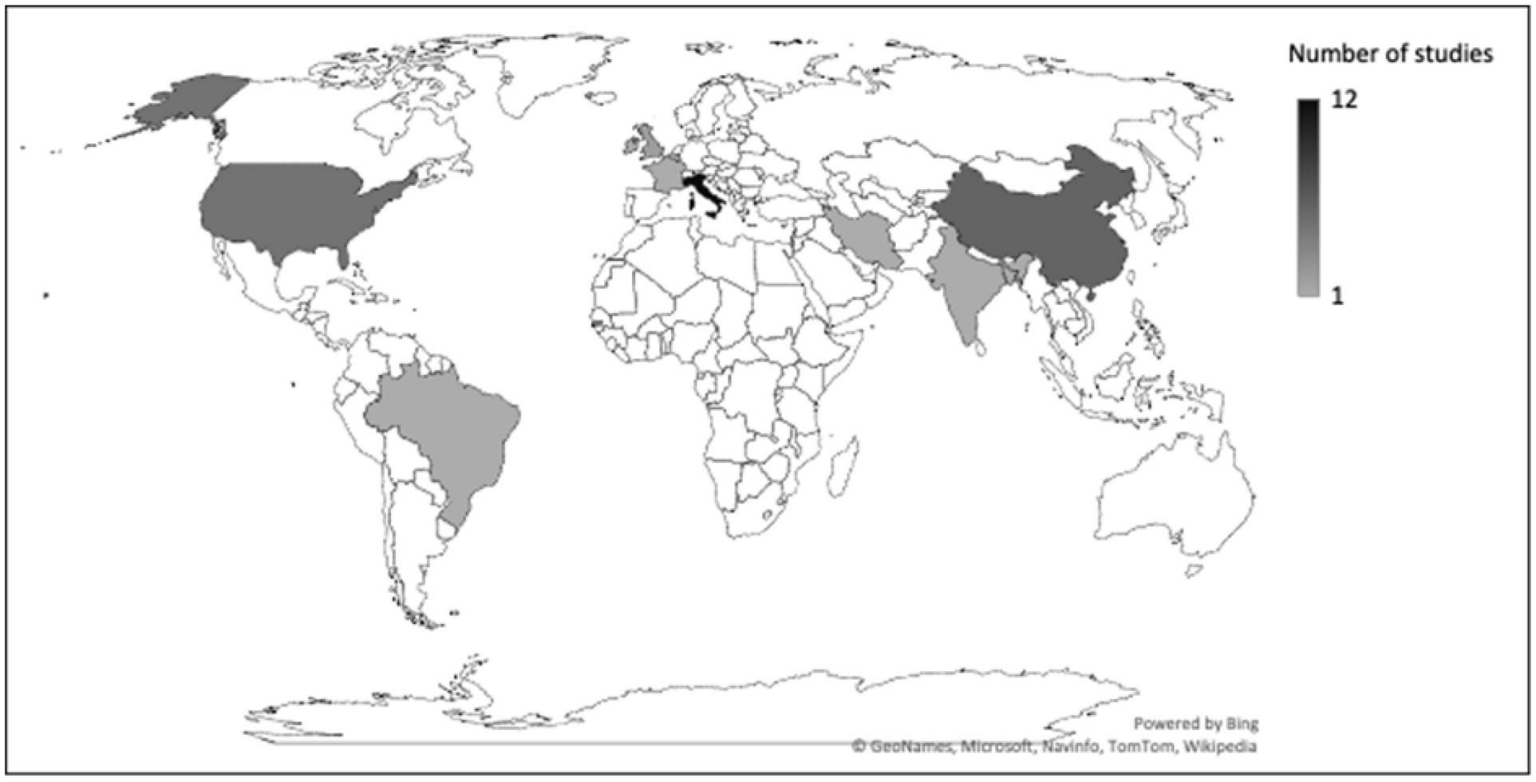
Origin and number of studies participating data in this systematic review.

**Figure 3.**
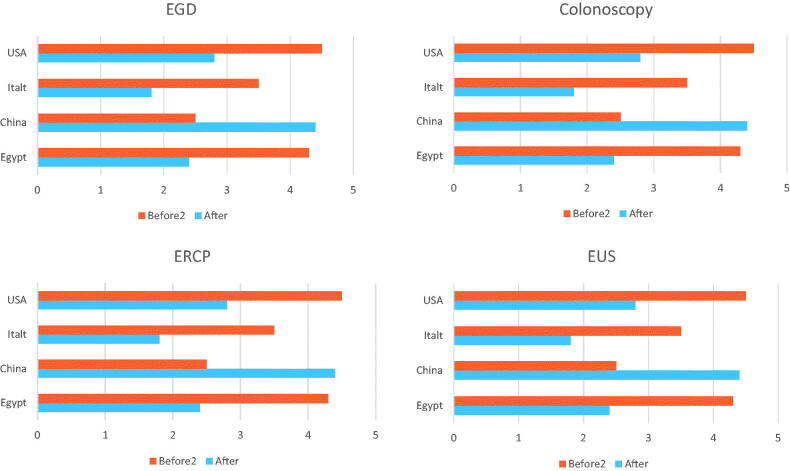
Types and relative volume reduction of different endoscopic procedures performed during COVID-19 pandemic in different continents compared to data before the pandemic (data from 31 centres. Each continent is represented by data form one big country).

The origin and the number of studies participating in this systematic review are presented in Figure 2. There was a discrepancy in the numbers and types of procedures reported to be done during the pandemic with more reports coming from Europe than rest of the world and with esophagogastroduodenoscopy (EGD), colonoscopy and endoscopic retrograde cholangiopancreatography (ERCP) being the most commonly performed procedures. Types and relative volume of different procedures performed during COVID-19 pandemic in different continents (data from 31 centres) are detailed in Figure 3.

### Endoscopy practice

#### Changes in endoscopy practice

The majority of the included studies provide real life data during the peak of the pandemic months March and April. Representatives from different endoscopy units and individual endoscopists confirmed the drastic reduction of all GI endoscopy procedures (total capacity) that ranged from 75% to almost 100% due to adoption of strict triage and prioritization regulations. Minority of centres reported continuation of the regular capacity [1,6,7]. Furthermore, the diagnostic rates of the performed routine endoscopies were significantly higher in many reports [8,9].

#### Compared to previous months due to prioritization

For Emergency endoscopy like upper GI bleeding and foreign body ingestion reports from France and Italy [8,9] showed marked decrease while contradictory reports from different parts of Italy showed no significant changes from the previous year (2019) rates [10]. This was attributed to their rapid adoption of strategies to accomplish these indications as emergencies that should not be deferred.

#### Gastrointestinal cancer screening

Colorectal cancer (CRC) screening was markedly affected. Target population for CRC screening were rescheduled and delayed by months. This resulted in reduction of the rate of the newly diagnosed GI cancers [11], but that was on the expense of the cancer grading. Minority of centres in Italy continued CRC screening [6].

### Infection and infection control

#### Precautions before endoscopic procedures (Table 2)

Out of the included studies, 17 reported performing triage by history taking for patients before endoscopy [1,7,10,12–24]. Only two studies [13,25] performed full assessment before endoscopy by complete blood count (CBC), chest X-ray (CXR), Computed Tomography on chest (CT Chest) and Polymerase Chain Reaction (PCR) testing for SARS-CoV-2 RNA while three studies checked patients by PCR and CT only [10,26,27]. Twelve studies relied only on PCR testing for triaging patients [7,9,16,18,24,28–34]. Two studies used both CBC and PCR testing for patient assessment [1,32]. One study used CBC, CT and PCR [17] and one study used CBC and CXR [12]. Clinical assessment of symptoms and temperature measurement were used by Navaneethan et al. [39], while Medas et al. [42] used a questionnaire as an initial triage.

#### Personal protective equipment (PPE) and staff shortage

Endoscopy procedures are aerosol generating and hence there is usually a real risk of infection among the staff. This was realized in the published literature and consequently PPE wearing was recommended by 28 studies [1,8–14,16,17,19–24,26,28–33,36–39]. Fifteen, studies reported PPE shortage [4,7,11,12,15,16,20,24,31,33,34,38,40–43] especially in the N95 masks, while staff shortage was reported in only nine studies [1,16,29–31,33,34,41,44], the frequency of shortage ranged between 49.02% [1] to 68.8% [16]. Staff reallocation to take part in the hospital fight against the COVID-19 was documented by 13 studies [11–13,29–31,33,37,45–49] (Supplementary Table 1). The reallocation involved both the endoscopists and nursing staff. For physicians one report from Italy documented that all physicians and surgeons switched their daily tasks to become temporary ICU and infectious diseases specialists [10].

#### Post endoscopy staff and patients, infection

Thirteen studies performed post-procedure screening for COVID-19 infection [1,12,13,15,17,19,21,29,30,32,36,37,45] (Supplementary Table 1). Post-procedure staff infection ranged from 0% in several studies [13,17,37,45], up to 30.5% in Alboraie et al. study [1], while post-procedure patient infection was reported by only five studies [1,14,17,32,36], and ranged from 0.64% in Dioscoridi and Carrisi [17] study up to 25.6% in Alboraie et al. study [1].

### Impact of COVID-19 on individual endoscopy types

#### Reduction in procedure volume

Data discussing the impact of the pandemic on individual endoscopy types retrieved from the literature is heterogeneous, either due to heterogenous reporting of different endoscopic procedures or due to stoppage of service in many gastrointestinal endoscopy units around the globe. In comparison to 2019, it is obvious that reduction in all endoscopic procedures was the trend during the COVID-19 pandemic; the reduction percent was variable between different centres. Al Mahtab et al. [12] reported 96% reduction in routine endoscopy service compared to the period before the pandemic. Centres continued to provide routine endoscopy service reported about 40% to 85% reductions. For upper endoscopy one study [11] reported reduction of the number of upper endoscopy procedures from 48302 in 2019 to 8878 in 2020 (81% reduction), the same study reported reduction in colonoscopy procedures from 29522 in 2019 to 4909 in 2020 (83.4% reduction). Reductions in ERCP procedures were elucidated by Alboraie et al. [1] in a multinational study showing reductions from 1612 in 2019 to 699 in 2020 (56.6% reduction). Endoscopic ultrasound (EUS) procedures were the least affected, in Huang et al. [23] and Alboraie et al. [1] studies, EUS procedures reductions were from 1114, 1064 in 2019 to 138, 329 in 2020, (87.6% and 69% respectively).

Not only routine procedures (EGD, Colonoscopy, ERCP and EUS) were reduced, but also urgent procedures were affected during the lockdown period as demonstrated by D'Ovidio et al. [8] and Salerno et al. [43], that is why time sensitive procedures were re-considered by many centres and many renewed endoscopists [1].

It was obvious from the published data that reductions in the individual endoscopic procedures was a global concern. Data from the Americas, Europe, Asia and Africa represented by data from USA, Italy, China and Egypt were consistent in this point although with different percentages (Figure 3).

#### Impact on the findings

Although endoscopic services were reduced, significant endoscopic findings were detected as reported by D'Ovidio et al. [8] and Salerno et al. [43] with increasing diagnostic yield of urgent endoscopy (Table 3). D'Ovidio et al. [8] reported higher frequency of impacted food bolus, bleeding angiodysplasia, Dieulafoy’s lesions and bleeding gastroduodenal ulcers (10/13 patients) that required blood transfusion and endoscopic interventions (in 50% of cases) using metallic clipping or hemospray than the comparable period before the pandemic.

**Table 3.** Diagnostic yield of urgent upper endoscopy during lockdown period in comparison to the previous year.

	Salerno et al. [43]		D'Ovidio et al. [8]	
	Before	During	Before	During
Esophago-gastro-duodenoscopy positive for findings (no., %)				
NVUGB	345 (56.1%)	240 (62.6%)		
VUGB	105 (17%)	56 (14.6%)		
Foreign body	84 (13.6%)	36 (9.4%)		
Caustic ingestion	24 (4%)	11 (3%)		
Others	57 (9.3%)	40 (10.4%)		
Severity of bleeding endoscopic stigmata (Forrest classification)				
III Clean‐based ulcer			7 (64%)	3 (30%)
IIc Flat pigmented haematinic			0	2 (20%)
IIb Adherent clot			3 (27%)	0
IIa Non‐bleeding visible vessel			0	3 (30%)
Ib Active oozing			1 (9%)	2 (20%)

## Discussion

The COVID-19 pandemic implements special situation over the world. A lot of changes have been taken in endoscopy practice to avoid infection transmission in parallel to keep providing the patient best care. In this review we focussed on the impact of COVID-19 pandemic on GI endoscopy practice. The included studies represent the data during the complete lockdown months of pandemic regarding procedure volume capacity in comparison to pre pandemic, infection control precautions, PPE and staff shortage.

Regarding changes in endoscopy practice, most of the included studies reported a significant reduction in total capacity (75% −100%) of all types of endoscopy procedures compared to 2019 and they attributed it to prioritization regulations. According to this special situation most of endoscopy centres worldwide rescheduled to receive emergency cases only that was recommended by most of the international GI endoscopy societies [50,51].

Unfortunately, only Repici et al. [6] reported CRC screening continuation in contrary to other included studies that followed prioritization regulations and deferred the elective procedures including screening. In this context it was detected that some studies showed 84%, 72% reduction in CRC screening in USA and UK, respectively [52,53]. However, recommendations of endoscopy societies to evaluate and individualize such patients case by case to not lose the best management if the diagnosis is delayed [54].

All the included studies in this review followed the GI societies guidance regarding the precautions that have been taken pre-procedure using different methods for patients triaging. According to our results minority of the included studies [13,25–27] reported the use of laboratory, radiological and serological assessment of the patient before the procedure. It was clear that, most of the included centres used the least coasty method such as history from the patient, X-ray or only PCR testing. Accordingly, it indicates that the different methods used for patients triaging was mostly related to the testing ability and the availability in each centre.

Almost, most of the national and international precautionary measures endorse the importance of PPE wearing to the staff before the procedure which was already reported in most of our included studies. However, the reported frequency of staff shortage ranged from 49.02% to 86.8%, where it was attributed to staff reallocation in the hospitals dealing with infected patients with COVID-19.This shortage in staff or in PPE was one of the important impacts of pandemic as it is reflected on endoscopy practice causing marked reduction in procedure volume.

Few included studies in our review reported post-procedure screening for COVID-19 infection among the staff and the patients. Post-procedure staff infection ranged 0%−30.5% and patient infection ranged 0.64%−25.6%, this low percentage may be related to the successful adherence to general precautionary measures. Nevertheless, this percentage may not be expressive to the real percentage of infected staff and patients as it was mentioned in few studies that was eligible to be included in our review.

Finally, it was surprisingly that two studies [8,43] reported an increase in the diagnostic yield for emergency cases. We do not have exact interpretation to such finding, but it may be attributed to the low numbers of the examined cases that allow the endoscopist to be more focussed. Furthermore, deferring the non-urgent procedures and focussing on highly selected cases probably increased tis diagnostic yield.

Limitations: The heterogeneity of included studies in the study designs, concerned items that they took up and rapidly conducted and published studies. A lot of factors affect the changes of endoscopy practice worldwide during the pandemic including geographic distribution and risk of infection, ability and availability of PPE, working staff and patient triage between different countries and application of different institutional policies. All of that contributing to studies heterogeneity. Our review included the studies that limited to the peak months of COVID 19 infection where endoscopy practice markedly affected, and we did not evaluate the afterward changes in different phases of COVID-19 pandemic.

Furthermore, it is clear from the data presented here that a wide gap exists between data from Europe and other parts of the world. It seems that, it may be related to many issues including the rapid large scale spread of the infection among European population in addition to the effective, rapid, multicentre publications from many endoscopy units in Europe in comparison to other parts of the word.

## Future and recommendations

Endoscopy practice is probably not going to be the same after the COVID-19 pandemic. As we have discussed, there have been several changes to adapt to the crisis and those changes have been quite heterogeneous even though the pandemic had recognizable similar phases in all countries. Some guidelines had been issued regarding the use of PPE and the treatment of the emergency patients and the few outpatients that are being assessed worldwide.

Now, is the time to plan, we need better guidelines based now on the evidence that has been produced in the last year to continue the usual indications for gastrointestinal endoscopy that were in place before the pandemic.

Also, we will have to consider the changes that will come after the vaccination that has just started worldwide and how the different waves of the pandemic and the variants of the virus will affect the next steps.

We recommend using this study as the basis for new studies that will take all new issues into account and help develop global guidelines for the new endoscopy practice.

## Conclusion

Our systematic review provides an overview about the major changes that happened in the endoscopy practice in the era of COVID-19 pandemic especially in lockdown months. The main outcomes from this review are rescheduling of endoscopy procedures to be suitable with the situation of COVID-19 pandemic in each country. Also, the endorsement of the importance of PPE use for health care workers and screening of COVID-19 infection pre-procedure. It was a good lesson to be adaptive on the changes that can happen in the future if we faced such an outbreak again.

## Supplementary Material

Supplemental Material

## Data Availability

The data associated with this review is available from the corresponding author upon request.
