# Supplementary material for: Effect of COVID-19 on gastrointestinal endoscopy practice: a systematic review
Source: Ann Med. 2022 Nov 11;54(1):2875–84. doi: 10.1080/07853890.2022.2133163 (PMC9665076; doi:10.1080/07853890.2022.2133163)
Supplement: Supplemental Material [file IANN_A_2133163_SM5155.docx]

**Supplementary Table 1:** focusing the use of protective equipments and staff condition during the COVID-19 pandemic

| **Study** | **PPE wearing** | **Post procedure screening** | **PPE shortage** | **Staff shortage** | **Staff Reallocation** |
| --- | --- | --- | --- | --- | --- |
| Al Mahtab, et al. | YES | Yes | YES | NO | YES |
| Alboraie, et al. | YES (for confirmed cases in 83.5-90.9%) | YES (in 18.3%) |  | YES(decreased by49.02%) |  |
| Alessandro et al. | YES(PPE, with gowns, googles, only surgical masks were available for most of the procedures in Northern Italy in the study period) | YES |  |  |  |
| An, et al | YES | YES | NO | NO | YES |
| Aurelio, et al | YES | NA |  |  |  |
| Becq A, et al | YES | NO | NO | NO | NO |
| Chen, et al | YES | NA |  |  |  |
| Dioscoridi, et al | YES |  |  |  |  |
| D'Ovidio, et al | - | - | - | - | - |
| Ebigbo, et al | YES | NO | YES |  |  |
| Elli, Luca, et ali |  |  |  |  |  |
| Forbes, et al |  | YES (only in 28%) | YES |  |  |
| Garbe, et al | YES |  | YES( 81.3%) | YES(68.8% of endoscopy units expected staff shortages during the pandemic) |  |
| Gianpiero et al | YES | Not mentioned |  | YES | YES |
| Goenka et al | YES  (74.7 % of endoscopistsused N95 or similar masks, 74.2 % used a face shield or other protective eyewear, 49.2 % used complete PPE). |  |  |  |  |
| Huang, et al | YES | YES | NO | YES | YES |
| Ikehara et al |  |  |  |  |  |
| Khamaysi, I. and S. Michlin |  |  |  |  |  |
| Kim, et al | YES | YES |  |  |  |
| Kushnir et al |  |  |  |  |  |
| Lamazza, et al |  | YES |  |  |  |
| Lauro, et al | YES |  |  |  | YES (All physicians and surgeons switched their daily tasks to become temporary ICU and infectious disease specialists) |
| Liu, et al | YES | 0 | 0 | 0 | 0 |
| Lui, et al | YES | 0 | 0 | 0 | 0 |
| Lui, et al | 0 | 0 | 0 | 0 | 0 |
| Mahadev et al | YES | NA | YES | NA | YES |
| Mahadev, et al | YES | 0 | YES | 0 | YES(in 53%) |
| Maida et al | YES |  | Yes |  |  |
| Manes et al | NA | NA | NA | YES | YES |
| Manes, et al | 0 | 0 | YES | YES | YES |
| Marasco et al | NA | NA | YES | NA | YES |
| Marcello, M. | 0 | 0 | 0 | 0 | 0 |
| Matthe et al | Not mentioned | Not mentioned | YES | Not mentioned | YES |
| Moreels, T. G. | YES | 0 | YES | YES | YES |
| Navane et al | YES (N 95 ) | YES |  |  |  |
| O’Grad et al | YES |  |  |  |  |
| Ovidio, et al | YES |  |  |  |  |
| Parasa, et al | YES (N95/ powered air-purifying respirator (PAPR) in 78.6% of cases, surgical masks in 65.9%, gloves in 96%, gown in 92.1%, and goggles in 83.7%). |  |  |  |  |
| Renato et al | YES | Not mentioned | NO | NO | NO |
| Repici, et al |  | YES |  |  | YES (65.9% of endoscopy units, endoscopists were located to other hospital departments, In 31 (75.6%) endoscopy units nurses were relocated to other hospital departments) |
| Salerno, et al | Not mentioned | Not mentioned | Not mentioned | Not mentioned | Not mentioned |
| Sharareh et al | YES | NO | YES | YES | YES |
| Sobani, et al | Not mentioned | Not mentioned | Not mentioned | Not mentioned | Not mentioned |
| Valeria et al | YES | YES | Not mentioned | Not mentioned | Not mentioned |
| Belle et al | NO | NO | YES | YES |  |
| di Pietro,et al | Not mentioned | Not mentioned | Not mentioned | Not mentioned | Not mentioned |
| Zorniak, et al | YES | NA | YES |  |  |
| PPE (Personal protective Equipment) | | | | | |
